# Methods for Separation and Characterization of Extracellular Vesicles: Results of a Worldwide Survey Performed by the ISEV Rigor and Standardization Subcommittee

**DOI:** 10.3390/cells9091955

**Published:** 2020-08-25

**Authors:** Felix Royo, Clotilde Théry, Juan M. Falcón-Pérez, Rienk Nieuwland, Kenneth W. Witwer

**Affiliations:** 1 Center for Cooperative Research in Biosciences (CIC bioGUNE), Basque Research and Technology Alliance (BRTA), Exosomes Laboratory, 48160 Derio, Spain; froyo.ciberehd@cicbiogune.es; 2 Centro de Investigación Biomédica en Red de Enfermedades Hepáticas y Digestivas (CIBERehd), 28029 Madrid, Spain; 3 Institut Curie, INSERM U932, PSL Research University, 75005 Paris, France; clotilde.thery@curie.fr; 4 IKERBASQUE, Basque Foundation for Science, 48013 Bilbao, Spain; 5 Laboratory of Experimental Clinical Chemistry, Department of Clinical Chemistry, Amsterdam UMC, Location AMC, University of Amsterdam, 19268 Amsterdam, The Netherlands; 6 Vesicle Observation Centre, Amsterdam UMC, Location AMC, University of Amsterdam, 19268 Amsterdam, The Netherlands; 7 School of Medicine, Departments of Molecular and Comparative Pathobiology and Neurology, Johns Hopkins University, Baltimore, MD 21205, USA

**Keywords:** extracellular vesicles, exosomes, microvesicles, microparticles, methodology, quality control, separation, isolation, characterization, biobanks, standardization

## Abstract

Research on extracellular vesicles (EVs) is growing exponentially due to an increasing appreciation of EVs as disease biomarkers and therapeutics, an expanding number of EV-containing materials under study, and application of new preparation, detection, and cargo analysis methods. Diversity of both sources and methodologies imposes challenges on the comparison of measurement results between studies and laboratories. While reference guidelines and minimal requirements for EV research have achieved the important objective of assembling community consensus, it is also essential to understand which methodologies and quality controls are currently being applied, and how usage trends are evolving. As an initial response to this need, the International Society for Extracellular Vesicles (ISEV) performed a worldwide survey in 2015 on “Techniques used for the isolation and characterization of extracellular vesicles” and published the results from this survey in 2016. In 2019, a new survey was performed to assess the changing state of the field. The questionnaire received more than 600 full or partial responses, and the present manuscript summarizes the results of this second worldwide survey. The results emphasize that separation methods such as ultracentrifugation and density gradients are still the most commonly used methods, the use of size exclusion chromatography has increased, and techniques based on tangential flow and microfluidics are now being used by more than 10% of respondents. The survey also reveals that most EV researchers still do not perform sample quality controls before or after isolation of EVs. Finally, the majority of EV researchers emphasize that separation and characterization of EVs should receive more attention.

## 1. Introduction

Studies of extracellular vesicles (EVs) in health and disease have increasingly confirmed the biological relevance of these membrane-delimited particles. The resulting groundswell of interest in the EV field has provoked an influx of new scientists, who bring fresh ideas to EV research. At the same time, the combination of rapid progress and incomplete penetrance of established experience has led to a certain amount of misunderstanding, miscommunication, and publication of less than rigorous results, in turn rousing continuous scientific debate and presenting opportunities for improved sharing. Since its founding in 2012, the International Society for Extracellular Vesicles (ISEV) has sought to seize these opportunities, expending extensive efforts to increase rigor and reproducibility. These initiatives have included the founding and evolution of a journal, the *Journal of Extracellular Vesicles* [1], and producing guidelines [2], position papers [3,4,5,6], dedicated courses during ISEV educational days, and massive open online courses (MOOCs) aimed at reaching general scientists interested in EV research. Both MOOC I [7] and MOOC II are freely available through Coursera (www.coursera.org/) and at the ISEV homepage (www.isev.org). Finally, ISEV announced the establishment of a Rigor and Standardization subcommittee at the annual ISEV meeting in Kyoto, Japan, in 2019 (ISEV2019).

An important part of the ISEV efforts for rigor and standardization is taking a regular “pulse” of the field, to understand which techniques are in use and where new developments might be needed. In 2016, Gardiner et al. published the results of a first worldwide survey on “Techniques used for the isolation and characterization of extracellular vesicles”. Organized by ISEV, this survey elicited 196 responses from researchers in 30 countries [8]. In addition, Soekmadji et al. performed a worldwide survey with a focus on RNA techniques, which was published in 2018 [2]. These two surveys were aimed at understanding how methods are being used in the EV field and at improving the comparison of results. With the formation of the ISEV Rigor and Standardization Subcommittee in 2019, a new survey was distributed, not only as a pulse-checking exercise, but also to identify expertise and interest for topic-specific task forces. Here, we report the results of the general survey. The presented data reflect the current status of the field, reveal how the field has evolved, and identify the next steps towards overcoming apparent challenges that are encountered in EV research.

## 2. The Survey

**Figure 1:**
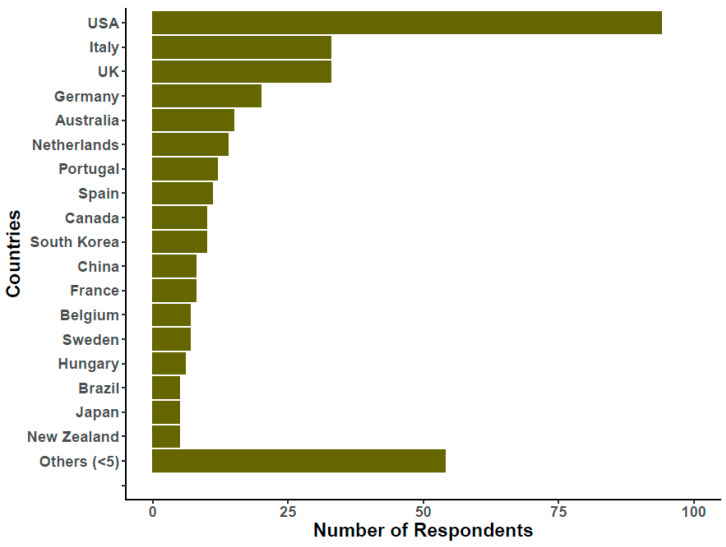
Number of respondents classified by the nationality of their institutional affiliation. The graph was generated from 357 responses.

Survey questions were generated by the ISEV Rigor and Standardization Subcommittee and are shown in the Supplementary Information (Supplementary Table S1). For comparison, questions from the original survey can be found in the Supplementary Material of Gardiner et al. [8]. The new survey was not simply a recapitulation of the earlier survey, so some questions and options were removed while others were added. The questions about the EV source and EV preparation methods were nearly identical, though, with only a few new options in 2019. ISEV advertised this effort in several electronic messages to the ISEV mailing list, on ISEV social media channels (e.g., Facebook and Twitter), and at the ISEV2019 annual meeting. The survey was opened on 24 March 2019 and closed in mid-August, 2019. A total of 620 full or partial submissions were recorded. Of these, 320 (approximately 52%) were complete (i.e., all questions answered). However, since it was not obligatory to answer all questions, all non-duplicated submissions were accepted for all questions. Overall, engagement was almost twice the level achieved in the previous, 2015 survey [8]. The number of respondents to each question is reported in the corresponding figure legends, and this number was used to calculate the indicated percentages for each answer. Of the respondents 85% were members of ISEV, and 86% belonged to academia. More than half were principal investigators, reflecting a strong level of engagement with the current survey at the senior level. Respondents also included industry participants (5%), government employees, and heads of flow cytometry or other core facilities or good manufacturing practice (GMP) units. As indicated by IP addresses, 40 countries on five continents were represented. The number of respondents per country is indicated (Figure 1). Notably, compared with the 2015 survey [8], there was increased participation of Asia-Pacific countries such China, South Korea, and Australia, as well as European countries such as Italy and Portugal.

## 3. Sources of Extracellular Vesicles

**Figure 2:**
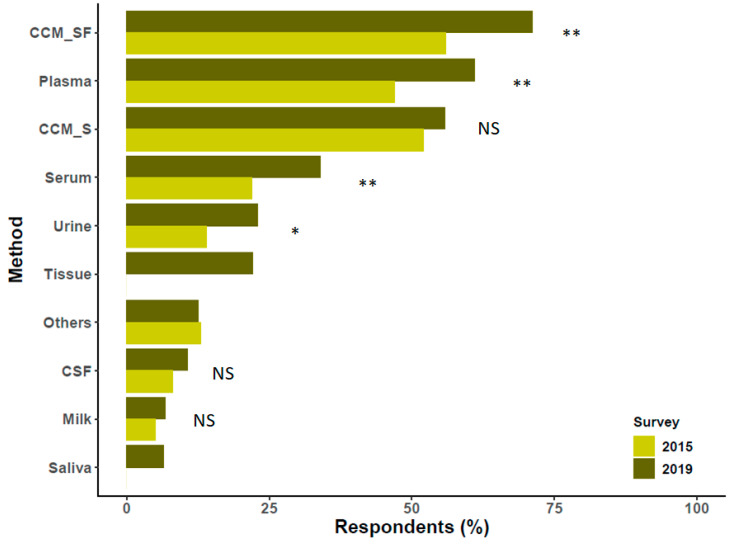
Sources of extracellular vesicles (EVs) and comparison with the report published previously by Gardiner et al. [8]. In the present survey (2019), there were 357 respondents to this question. Differences between proportions were tested by a z test, and *p* values were obtained by a chi-square test (NS, non-significant, * *p* < 0.05, ** *p* < 0.01. Missing bars: not queried in the 2015 survey). Please note that multiple answers were possible. Abbreviations: cell culture media enriched with serum (CCM_S); serum-free cell culture media (CCM_SF); cerebrospinal fluid (CSF).

The first scientific question assessed sources of EVs. The source material used by the largest percentage (almost 76% of 396 respondents) was cell culture-conditioned medium (CCM) without serum (Figure 2), and 59% used serum-containing CCM. In terms of biological fluids, 62% harvested EVs from blood plasma, and 34% used blood serum. Urine (24%) and cerebrospinal fluid (11%) were the next most utilized biofluids. Compared with the 2015 survey, use of saliva was now reported by 6%. Respondents named more than 15 additional biological or environmental fluids; of these, bronchoalveolar lavage, peritoneal fluid, and semen each received four mentions (around 1%). The overall results resembled those of the 2015 survey, but with blood plasma now as the second most utilized source instead of serum-containing CCM. Of the respondents 15% reported EV separation from biological tissues. Not all respondents specified the tissue type(s). However, brain tissue appeared to be used most commonly, followed by tumor tissues (various), placenta, and heart. There was a single mention of each of the following: bone marrow, eye, liver, mucosa (unspecified), pancreas, prostate, skin, thymus, Wharton’s jelly, and whole blood. Importantly, studies were not restricted to mammalian EVs. Worms (unspecified) and plant tissues (various) were also reported. Several respondents indicated that they studied parasites, but it was not clear if EVs were isolated from cultures (as we assumed for the purposes of analysis) or from whole parasites. We stress that, since this question was about source material only, we cannot say how these various materials were collected, processed, or stored prior to EV studies. More detailed information may be gathered in the future by source material-specific task forces of the ISEV Rigor and Standardization Subcommittee.

## 4. Preparation/Separation Methods

**Figure 3:**
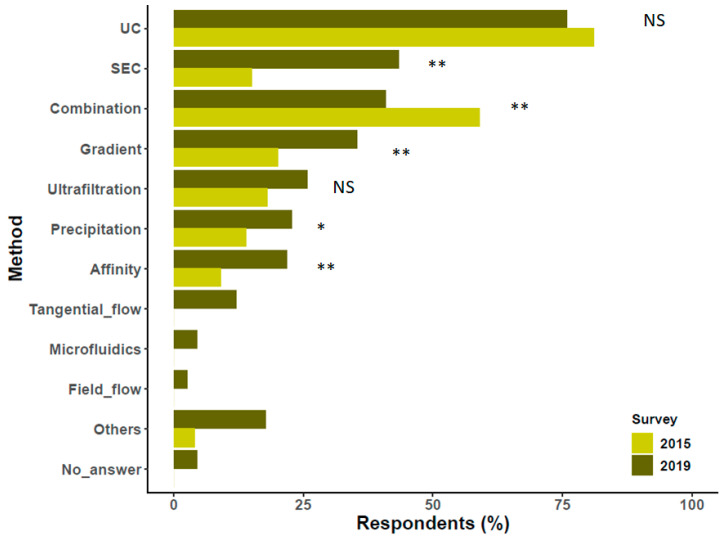
Methods used for EV separation compared with results of Gardiner et al. [8]. Results based on 357 responses. Differences between proportions were tested by a z test, and *p* values were obtained by a chi-square test (NS, non-significant, * *p* < 0.05, ** *p* < 0.01. Missing bars: not queried in the 2015 survey). Please note that multiple answers were possible. Affinity capture (Affinity), Combination of methods (Combination), Density gradient (Gradient), Field flow fractionation (Field_flow), Microfluidics (Microfluidics), Precipitation methods (Precipitation), Size exclusion chromatography (SEC), Tangential flow filtration (Tangential_flow), Ultracentrifugation (UC).

Although ultracentrifugation (UC), alone or combined with other techniques, is still the most commonly used EV separation method, the proportion of respondents who apply size exclusion chromatography (SEC) increased markedly since 2016 (Figure 3). Use of the other techniques did not change substantially. We would like to highlight that affinity techniques were used as often as precipitation methods. Furthermore, use of ultrafiltration did not increase to the same extent as other techniques. A sizable proportion of respondents embraced newer applications of tangential flow (12%), microfluidics (4%), and field flow fractionation (2%) to separate EVs or EV subpopulations.

## 5. Quality Control of EV Preparations: Relationship with Biobanking

**Figure 4:**
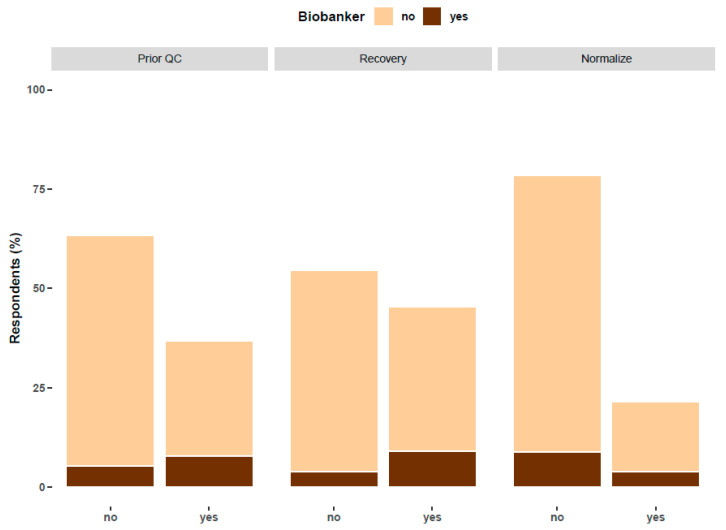
Differences in the application of different quality controls among researchers who prepare samples for biobanks (called “biobankers”) and those who do not. The question referenced as “Prior QC” reads, “Do you perform any form of quality sample control prior to EV separations?” The question referenced as “Recovery” reads, “Do you quantify recovery, specific activity, and/or contaminants in your concentrated or separated EV preparation?” and the question referenced as “Normalize” reads, “Do you normalize EV yield for any possible biological dilution of the biofluid?”. The percentage was calculated over 357, 341 and 326 respondents for each question, respectively.

The use of biobanks to store or share EVs is common to only a minority of EV researchers. About 25% of respondents reported using samples from biobanks, while a similar percentage prepared samples for biobanks. Since biobanking usually requires strict quality control (QC), relationships with biobanks may mean that quality control information and other metadata are more likely to be available for biobanked samples. The survey data supports this supposition. Overall, 63% of responding researchers did not perform a quality analysis of their samples prior to separation, 55% did not perform quantification of recovery versus contaminants, and 79% did not normalize according to the dilution of the biofluid of origin (Figure 4). However, of respondents who biobank (dark brown column, Figure 4), a majority (59%) quality controlled the EV source (odds ratio 3.2, 95% C.I. 2.055 < OR < 6.751), and 70% quantitated EV recovery (odds ratio 4.1, 95% C.I. 2.138 < OR < 8.198). Thus, researchers in contact with biobanks appear to give more attention to QC.

The survey results also revealed practices for source material QC (prior to EV separation) and EV recovery. Regarding QC prior to EV separation from blood or fractions thereof, hemolysis and platelet counting are the most commonly controlled parameters as observed previously [6], along with other blood chemistry parameters (i.e., hemoglobin). For cell culture, cell viability is often reported, as is the presence of specific cell/EV surface markers. To quantitate recovery and purity, those who work with blood plasma commonly use lipoproteins, immunoglobulins, and albumin as controls, while urine researchers look at combinations of parameters of total protein, Tamm-Horsfall protein (uromodulin), particle numbers, and RNA. Finally, those who wish to control for biofluid dilution usually track internal reference proteins from the biological system of interest (such as creatinine in urine or albumin in blood) or control for particle numbers prior to and after separation, taking in account the dilution factor.

## 6. EV Biomolecule Cargo Types of Interest

**Figure 5:**
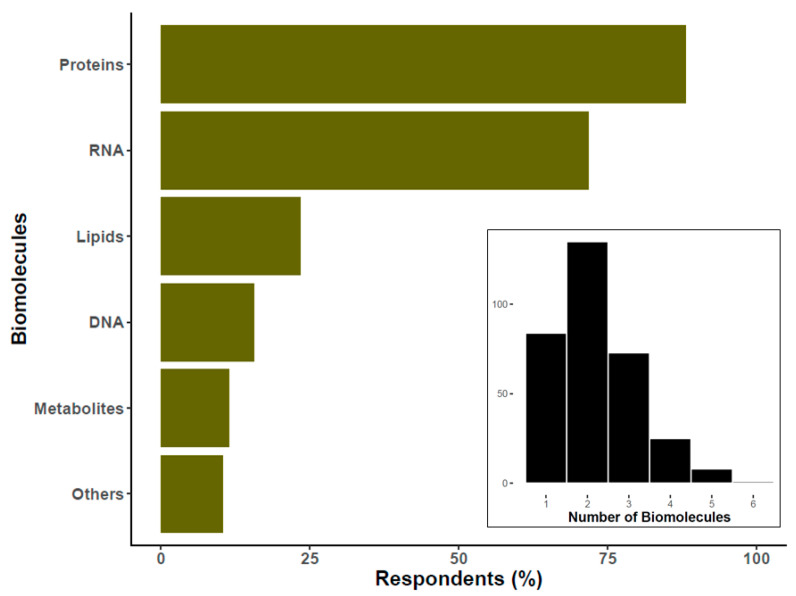
Percentage of researchers interested in specific types of EV-associated biomolecules. The histogram at the bottom of the graph reflects the number of researchers (*Y*-axis) that study the specified number of biomolecule classes (*X*-axis). For the main graph, the percentage was calculated over 326 respondents. Please note that this question had the possibility of multiple answers.

Most researchers specified two broad types of EV biomolecular cargo of interest, e.g., protein, DNA, RNA, lipids, etc.; see insert in Figure 5. Of the respondents 33% reported measuring or profiling three or more biomolecule categories. Among these, the most studied are proteins, followed by RNA (Figure 5). Almost 20% of researchers also mentioned DNA as a molecule of interest. We should clarify that, under the category of “others”, which allowed free entry of text, most respondents cited a particular protein or miRNA. A similar question from the 2015 survey focused on “downstream applications” rather than biomolecules of interest, so we do not compare answers in the graph. However, it is interesting to note that RNA assays were the predominant downstream assay in the 2015 results, whereas here, proteins seem to have overtaken RNA. Furthermore, lipids are a target of study for around a quarter of respondents, while “lipidomics” was a downstream assay for only 5% in 2015.

## 7. Increased Implementation and Diversity of Characterization Methods

**Figure 6:**
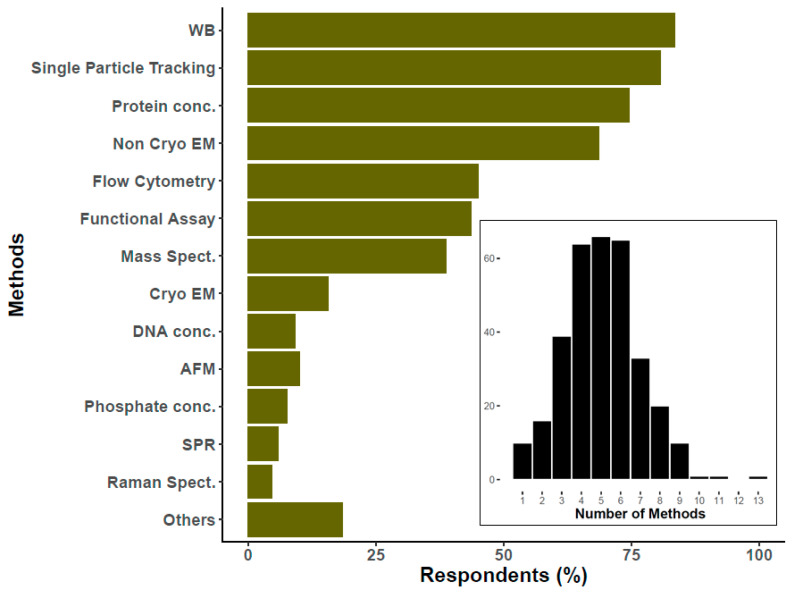
Percentage of researchers that use specific technique to characterize EVs. The histogram at the bottom of the graph reflects the number of researchers (*Y*-axis) that use the specified number of methods to characterize EVs (*X*-axis). For the main graph, the percentage was calculated over 326 respondents. Please notice that this question had the possibility of multiple answers. Atomic force microscopy (AFM), Cryogenic electron microscopy (Cryo EM), DNA concentration (DNA conc.), Flow cytometry, Functional assays (Functional Assay), Mass spectroscopy (Mass Spect.), others (including among others ELISA, Colorimetric nanoplasmonic, and Next Generation Sequencing), Particle tracking (Single Particle Tracking), Phosphate/phospholipids concentration (Phosphate conc.), Protein concentration (Protein conc.), Raman spectroscopy (Raman), Surface plasmon resonance (SPR), Transmission or scanning electron microscopy (Non Cryo EM), Western blotting (WB).

To characterize EVs, most researchers reported using Western blotting, single particle tracking, protein concentration, and electron microscopy (not including cryogenic electron microscopy (cryo-EM)) to characterize EVs (Figure 6). The next most widespread method, with the potential to provide either population-level or single-particle biochemical and physical information, is flow cytometry, also in agreement with the previous survey [8]. In the current survey, researchers were also asked about methodologies that address EV composition, such as mass spectrometry. Various approaches were reported to assess physical characteristics of single EVs (e.g., atomic force microscopy), biochemical composition (such as Raman spectroscopy), or identification/quantification of surface markers (for example, by surface plasmon resonance imaging). Altogether, these results show that respondents now use around four to six EV characterization methods (histogram Figure 6), whereas 60% of the 2015 respondents mentioned three or fewer. Indeed, for all methods queried in both the 2015 and 2019 surveys, usage appears to have increased; protein concentration measurements are two-fold what was reported in 2015 [8].

## 8. Normalization Methods for In Vitro and In Vivo Assays

**Figure 7:**
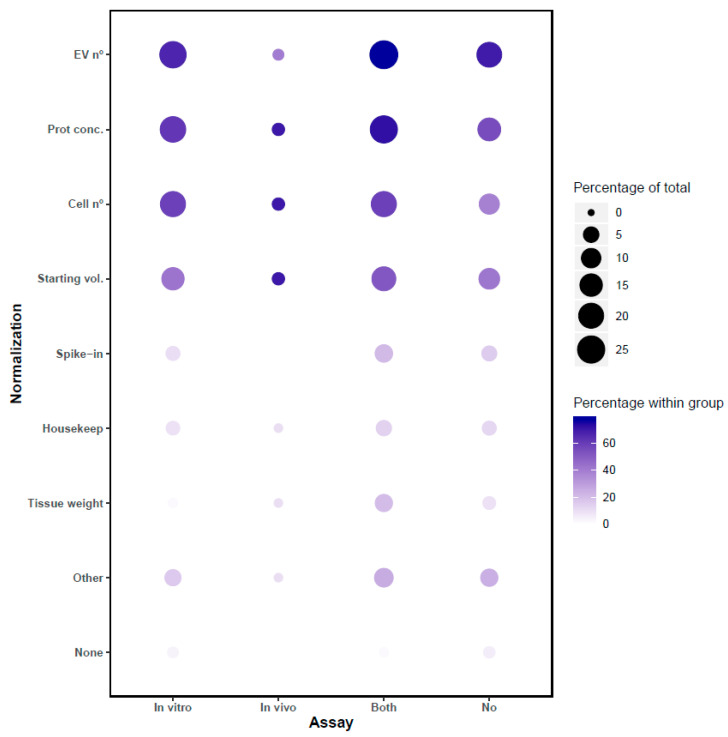
Techniques to normalize the use of EVs for downstream applications depend on whether the researchers perform in vitro assays, in vivo assays, both types of assays, or neither. Categories in the *Y*-axis are organized from more to less frequent normalization method in the in vitro group. Balloon size represents the percentage calculated using as total the number of respondents of this question (323). Balloon color intensity indicates the percentage calculated using the number of respondents within each group (111 only in vitro, 10 only in vivo, 111 perform both types of assays, and 91 none). Please note that this question had the possibility of multiple answers. Abbreviations: EV number (EV nº), Internal molecule (Housekeep), others (absorbance at 280 nm, lipid amounts, miRNAs, mixture of peptides, etc.), Producing cell number (cell nº), Protein concentration (Prot conc.), Spike-in molecule (Spike-in), Volume of medium or biofluid (Starting vol.) Tissue source weight (Tissue weight).

Controlling input of EVs for functional studies, whether in vitro or in vivo, is crucial for interpretation of results, so we sought to gather information on normalization in the 2019 survey. Here, 34% of respondents reported performing in vitro assays of EV function, while 34% performed both in vitro and in vivo, and 3% performed only in vivo assays. Of the researchers, 27% did not perform any kind of functional assay (in comparison with the 2015 survey, reported in vivo assay use has increased by more than 25%, while in vitro assay usage has stayed at about the same level [8]). To control input into functional assays, most respondents reported using “EV number” and “protein quantification”. Since normalization is assay-dependent, we also analyzed the data after stratifying for the different categories of functional assays (and none). As displayed in Figure 7, the most employed normalization methods are “EV number”, “protein concentration”, “number of cells”, and “starting volume prior to EVs isolation”. Internal housekeeping molecules or added spike-ins are also used, although less commonly than the above.

Regarding the differences between groups, it is clear that researchers who do not perform functional assays rely more on EV number and protein concentration, while those who perform in vivo assays rely less on particle counts. According to these results, in vivo assays may be perceived as demanding greater standardization, since among scientists who perform in vivo assays, less than 1% chose the option “none” for functional assays.

## 9. Conclusions and Final Remarks

The present survey provides a snapshot of the most common methods and practices of EV scientists, highlighting new trends in a field that is doubling the number of publications year over year. To summarize briefly and synthesize some conclusions include:

EV source: As in the study of Gardiner, et al. [8], cell culture-conditioned serum-free medium is the most common source of EVs. Importantly, while serum-free culture may seem ideal for EV studies, it should be remembered that additives in serum-free medium may also contain presumed EV cargo molecules [9], and that serum-free or EV-depleted serum conditions may affect quantitative and qualitative aspects of EV release [10,11]. Blood plasma has now overtaken serum-containing medium as the second most-used source of EVs [12]. Another biofluid, saliva, was not registered in the previous survey but is now studied by 6% of respondents, in line with recent indications of its versatility in biomarker applications [13,14]. Overall, the growing number of researchers sourcing EVs from biofluids relative to cell culture suggests a shift of the EV field towards more in vivo studies and clinical assays. Consistent with this, 10% of respondents reported contacts with regulatory agencies for the use of EVs in clinical trials. In the future, through material-specific task forces, the ISEV Rigor and Standardization team will seek to make recommendations about preanalytical variables and (perhaps more importantly) quality control measurements.

EV separation: Several years after the 2015 survey [8], ultracentrifugation remains the most common EV separation technique, despite potential drawbacks such as aggregation and incomplete separation [15,16]. However, there has been a marked growth in the use of gentler techniques such as SEC, tangential flow filtration, gradients, and affinity capture, consistent with the findings of a recent report [17]. This outcome suggests openness to new applications to achieve project-specific acceptable balances of yield, purity, and functionality, as indeed recommended by ISEV [2].

EV characterization: The trend in recent years is towards more characterization and more diverse types of measurement, consistent with the characterization recommendations of ISEV [2]. While the two most broadly applied characterization methods (Western blotting and single particle tracking) have not changed substantially since 2015, the relatively simple readout of protein concentration (#3) is now even more common than non-cryo EM (#4). Furthermore, a variety of newer methods, such as Raman spectroscopy and surface plasmon resonance (SPR), are nearing the application rate of atomic force microscopy (AFM), which holds steady at around 10%. A major finding is that most respondents routinely use 4–6 characterization techniques, up from an average of three as recorded previously [8]. While assessment of single markers (Western blot) will continue to be useful, the field should strive for wider adoption of techniques that provide maximum compositional and phenotyping data for each input.

What drives rigor and standardization? One of the novelties of this study arises from the questions about biobanks as a source and archive of EVs. It was previously predicted that the unique needs of EV biobanking would stimulate more standardization, including adoption of standard operating procedures [18]. In the present survey, we observed that “biobankers” are indeed ahead of the curve in adopting QC measures. At the same time, it is surprising that relatively few researchers perform QC of their EV source prior to separation. Similarly, common controls for protein contamination are often not performed. This lacuna may introduce problems, especially as the field moves from relatively well-behaved samples (such as cell culture-conditioned media) to biofluids, whose characteristics can differ drastically between individual donors. Anticipating this problem, Soekmadji et al., in their report on EV and exRNA mechanisms and standardization, pointed out that the wish-list of EV researchers included technique optimization, standardized protocol development, and enhanced exchange of knowledge [2]. Furthermore, Clayton et al. published a first roadmap towards collection, handling, and storage of extracellular vesicles from blood, in which the needs included education and quality control parameters [6]. The present survey as well as several recent publications emphasize these needs in EV composition and sample preparation methodology [19,20,21,22].

What about applications? Like the 2015 survey, this survey focused on methods, so we could not analyze trends in EV applications. However, in optional text submissions on in vivo assays, the most commonly mentioned systems/diseases were cancers (34%), skeletal muscle/tendon/bone (including arthritis; 11%), heart (9%), brain/spine (8%), autoimmune disease (7%), and infectious diseases (6%). These were followed by lung, blood/coagulation, and wound healing, all at around 5%. Diabetes, liver, pregnancy, aging, intestine, eye, and kidney were also mentioned. We stress that these results were collected from free-form answers, and that questions were not designed specifically to identify fields of study.

With the field of EVs still growing, and particularly as therapeutic uses of EVs become a reality, the challenges of rigor and standardization have come to the fore. By taking the pulse of community practice, the ISEV Rigor and Standardization survey of 2019 provides valuable information about current approaches, complementing the ISEV educational courses, position papers, and quality guidelines.

## Supplementary Materials

The original questions posted in the survey are collected in Supplementary Table S1. The following are available online at https://www.mdpi.com/2073-4409/9/9/1955/s1, Table S1: Survey.

## References

[1] Lötvall J., Rajendran L., Gho Y.-S., Théry C., Wauben M., Raposo G., Sjöstrand M., Taylor D., Telemo E., Breakefield X.O. (2012). The launch of Journal of Extracellular Vesicles (JEV), the official journal of the International Society for Extracellular Vesicles—About microvesicles, exosomes, ectosomes and other extracellular vesicles. J. Extracell. Vesicles.

[2] Soekmadji C., Hill A.F., Wauben M.H., Buzás E.I., Di Vizio D., Gardiner C., Lotvall J., Sahoo S., Witwer K.W. (2018). Towards mechanisms and standardization in extracellular vesicle and extracellular RNA studies: Results of a worldwide survey. J. Extracell. Vesicles.

[3] Lener T., Gimona M., Aigner L., Börger V., Buzas E., Camussi G., Chaput N., Chatterjee D., Court F.A., Del Portillo H.A. (2015). Applying extracellular vesicles based therapeutics in clinical trials—An ISEV position paper. J. Extracell. Vesicles.

[4] Mateescu B., Kowal E., Van Balkom B.W.M., Bartel S., Bhattacharyya S.N., Buzás E.I., Buck A.H., De Candia P., Chow F.W.-N., Das S. (2017). Obstacles and opportunities in the functional analysis of extracellular vesicle RNA—An ISEV position paper. J. Extracell. Vesicles.

[5] Russell A.E., Sneider A., Witwer K.W., Bergese P., Bhattacharyya S.N., Cocks A., Cocucci E., Erdbrügger U., Falcon-Perez J.M., Freeman D.W. (2019). Biological membranes in EV biogenesis, stability, uptake, and cargo transfer: An ISEV position paper arising from the ISEV membranes and EVs workshop. J. Extracell. Vesicles.

[6] Clayton A., Boilard E., Buzás E.I., Cheng L., Falcón-Perez J.M., Gardiner C., Gustafson D., Gualerzi A., Hendrix A., Hoffman A. (2019). Considerations towards a roadmap for collection, handling and storage of blood extracellular vesicles. J. Extracell. Vesicles.

[7] Lässer C., Théry C., Buzás E.I., Mathivanan S., Zhao W., Gho Y.S., Lötvall J. (2016). The International Society for Extracellular Vesicles launches the first massive open online course on extracellular vesicles. J. Extracell. Vesicles.

[8] Gardiner C., Di Vizio D., Sahoo S., Théry C., Witwer K.W., Wauben M., Hill A.F. (2016). Techniques used for the isolation and characterization of extracellular vesicles: Results of a worldwide survey. J. Extracell. Vesicles.

[9] Auber M., Fröhlich D., Drechsel O., Karaulanov E., Krämer-Albers E.-M. (2019). Serum-free media supplements carry miRNAs that co-purify with extracellular vesicles. J. Extracell. Vesicles.

[10] Kowal J., Arras G., Colombo M., Jouve M., Morath J.P., Primdal-Bengtson B., Dingli F., Loew D., Tkach M., Théry C. (2016). Proteomic comparison defines novel markers to characterize heterogeneous populations of extracellular vesicle subtypes. Proc. Natl. Acad. Sci. USA.

[11] Eitan E., Zhang S., Witwer K.W., Mattson M.P. (2015). Extracellular vesicle–depleted fetal bovine and human sera have reduced capacity to support cell growth. J. Extracell. Vesicles.

[12] Coumans F.A., Brisson A.R., Buzás E.I., Dignat-George F., Drees E.E., El Andaloussi S., Emanueli C., Gąsecka A., Hendrix A., Hill A.F. (2017). Methodological Guidelines to Study Extracellular Vesicles. Circ. Res..

[13] Han Y., Jia L., Zheng Y., Li W. (2018). Salivary Exosomes: Emerging Roles in Systemic Disease. Int. J. Boil. Sci..

[14] Chiabotto G., Gai C., Deregibus M.C., Camussi G. (2019). Salivary Extracellular Vesicle-Associated exRNA as Cancer Biomarker. Cancers.

[15] Webber J., Clayton A. (2013). How pure are your vesicles?. J. Extracell. Vesicles.

[16] Erdbrügger U., Rudy C.K., Etter M.E., Dryden K.A., Yeager M., Klibanov A.L., Lannigan J.A. (2014). Imaging flow cytometry elucidates limitations of microparticle analysis by conventional flow cytometry. Cytom. Part A.

[17] (2019). Trends in Extracellular Vesicle Research. https://www.beckman.es/resources/research-areas/nanoscale/ev-report.

[18] Mora E.M., Álvarez-Cubela S., Oltra E. (2015). Biobanking of Exosomes in the Era of Precision Medicine: Are We There Yet?. Int. J. Mol. Sci..

[19] Zhang H., Lyden D. (2019). Asymmetric-flow field-flow fractionation technology for exomere and small extracellular vesicle separation and characterization. Nat. Protoc..

[20] Jeppesen D.K., Fenix A.M., Franklin J.L., Higginbotham J.N., Zhang Q., Zimmerman L.J., Liebler D.C., Ping J., Liu Q., Evans R. (2019). Reassessment of Exosome Composition. Cell.

[21] Bebelman M.P., Bun P., Huveneers S., Van Niel G., Pegtel D.M., Verweij F.J. (2019). Real-time imaging of multivesicular body–plasma membrane fusion to quantify exosome release from single cells. Nat. Protoc..

[22] Leidal A.M., Huang H.H., Marsh T., Solvik T., Zhang D., Ye J., Kai F., Goldsmith J., Liu J.Y., Huang Y.-H. (2020). The LC3-conjugation machinery specifies the loading of RNA-binding proteins into extracellular vesicles. Nat. Cell Biol..

