# Supplementary material for: Methods for Separation and Characterization of Extracellular Vesicles: Results of a Worldwide Survey Performed by the ISEV Rigor and Standardization Subcommittee
Source: Cells. 2020 Aug 25;9(9):1955. doi: 10.3390/cells9091955 (PMC7563174; doi:10.3390/cells9091955)
Supplement: Supplementary file 1 [file cells-09-01955-s001.pdf]

Survey

We are happy to introduce the new ISEV Subcommittee on scientific rigor and reproducibility. The main goal of this survey is to identify ISEV members who are willing to contribute to or lead task forces to improve the comparison and reproducibility of our measurement results on EVs. In turn, improved comparison and reproducibility will facilitate implementation of EV-based research for clinical and non-clinical applications. The outcome of this survey will be presented at the ISEV 2019 meeting in Kyoto.

Contents

Survey 1

Page: Contact information 3

Page: starting materials and pre-analytical variables 4

Page: EV preparation 5

Page: EV characterization 6

Page: reference materials, calibration, and normalization 7

Page: EV functional studies 8

Page: regulatory affairs 9

Page: standardization studies and products 10

Page: Task force volunteers 11

Contact information

1. Your first (given) name
2. Your last (family) name
3. Your email address (by giving your email, you agree that ISEV may contact you about the survey results)

Starting materials and pre-analytical variables

4. What is your starting material in EV studies? (tick all that apply)
    - Blood
    - Cerebrospinal fluid
    - Conditioned cell culture medium (serum added)
    - Conditioned cell culture medium (serum-free)
    - Milk
    - Plasma
    - Saliva
    - Serum
    - Solid tissue
        - Comments box
    - Urine
    - Other
        - Comments box

5. Are you using samples from biobanks?
    - Yes/No
        - Comments box (What type and how many specimens?)

6. Are you preparing samples for biobanks?
    - Yes/No
        - Comments box (What type and how many specimens?)

7. Do you perform quality sample control prior to EV separations? For example, for plasma, do you measure residual platelets or haemolysis?
    - Yes/No
        - Comments box

EV preparation

8. Which techniques do you use for EV preparation? (tick all that apply)
    - Affinity capture methods
    - Density gradient
    - Field flow fractionation
    - Microfluidics
    - Precipitation
    - Size-exclusion chromatography
    - Ultracentrifugation
    - Ultrafiltration
    - Tangential flow filtration (TFF)
    - Combination of methods
    - Other, please specify
        - Comments box

9. Do you quantify recovery, specific activity and/or contaminants in your concentrated or isolated EV preparation? For example, THP in urine as a contaminant? Please specify the biofluid followed by a brief description.
    - Yes/No
        - Comments box (please specify)

EV characterization

10. Which of the following EV components do you study ? (tick all that apply)
    - DNA
    - Lipids
    - Metabolites
    - Protein
    - RNA
    - Other (please specify)
        - Comments box

11. How do you detect and characterize EVs? (tick all that apply)
    - Atomic force microscopy
    - Cryo-Electron microscopy
    - DNA concentration
    - Flow cytometry/nano-flow
    - Functional assay
    - Mass spectrometry
    - Particle tracking analysis (PTA, NTA)
    - (Tunable)-Resistive pulse sensing
    - Phosphate concentration (phospholipids)
    - Protein concentration
    - Raman spectroscopy
    - RNA concentration
    - Surface plasmon resonance
    - Transmission Electron Microscopy (negative staining) or other non-cryo electron microscopy
    - Western blotting
    - Other, please specify
        - Comments box

Reference materials, calibration, and normalization

12. Do you use a reference material as (internal) control for efficacy and reproducibility of EV preparation? (tick all that apply).
    - Aliquots of “normal” body fluids, please specify
        - ○ Comments box
    - Commercial EVs from external source, please specify
        - ○ Comments box
    - Commercial synthetic reference particles, please specify
        - ○ Comments box
    - Other, please specify
        - ○ Comments box

13. When you detect or characterize your EVs, do you perform any type of calibration for your instrument or system?
    - Yes (if yes, please indicate in the comments box which property is calibrated, and which reference material is used; e.g. flow rate of flow cytometry using reference particles, size for NTA using reference particles, etc.)
        - ○ Comments box
    - No

14. Do you normalize your EV yield for any possible biological dilution of the biofluid ? (For example, using creatinine to have into account urine dilution).
    - Yes/No (If yes please specify in the comment box)
        - ○ Comments box

15. Do you normalize your downstream results of functional and/or omics assays? (tick all that apply)
    - Yes/No (please specify)
        - ○ Comments
    - EV protein concentration
    - EV number
    - Internal molecules. Please indicate what molecule
        - ○ Comments box
    - Cell number
    - Spike-in molecule. Please indicate what molecule.
        - ○ Comments box
    - Tissue weight
    - Volume of medium or body fluid
    - Other
        - ○ Comments box

EV functional studies

16. Do you study function of EVs in vitro? In what assay(s)? Please provide comments and PMID(s) as appropriate).
    - Yes/No
        - Comments box
17. Do you study function of EVs in vivo? In what organism(s)? Please provide comments and PMID(s) as appropriate)
    - Yes/No
        - Comments box

Regulatory affairs

18. Do you have contacts with any regulatory agencies for clinical application of EVs?
    - Yes/No

19. If yes: If you have regulatory contacts for therapeutics, which contact, at what agency/agencies?
    - ○ Comments box

20. If yes: If you have regulatory contacts for biomarkers, which contact, at what agency/agencies?
    - ○ Comments box

Standardization studies and products

21. Do you participate (or have you participated) in (EV) standardization studies? Please tick all that apply and provide reference number(s) [PMID] in the comments box.
    - (Clinical) multi-centre studies
    - Comparison of techniques? (Please specify techniques)
        - Comments box
    - Comparison of physical EV properties? For example, the concentration of EVs in a provided sample, EV diameter, refractive index, fluorescence). (Please specify)
        - Comments box
    - Other
        - Comments box

22. Please rank the following as highest priority (1) to lowest priority (9), in your view, as topics of guidelines, reviews, methods, or position papers:
    - Biobanking
    - Biomarker development
    - Characterization technologies
    - Databases and knowledgebases
    - Navigating regulatory affairs (perhaps country/region-specific)
    - Reference materials and normalization approaches
    - Separation technologies
    - Specific EV sources (e.g., biofluids, tissue, culture medium)
    - Therapeutics development

Task force volunteers

On this page, please indicate your interest in contributing to a task force on up to three of the following topics by entering PMIDs to establish your expertise. A certain time commitment will be required if you are selected. If you have not entered your contact info on the first page, please go back; otherwise, we cannot follow up with you!

- Blood (plasma, serum)
    - Comments box (PMID)
- Cerebrospinal fluid
    - Comment box (PMID)
- Conditioned cell culture medium
    - Comments box (PMID)
- Milk
    - Comments box (PMID)
- Saliva
    - Comments box (PMID)
- Tissues
    - Comments box (PMID)
- Urine
    - Comments box (PMID)

- Biobanks and biobanking
    - Comments box (PMID)
- Biomarker development and navigating regulatory affairs for biomarkers
    - Comments box (PMID)
- Characterization technologies (to be determined later)
    - Which EV characterization technologies are mature enough to merit an ISEV guidelines or methods paper (list up to five with PMIDs to establish your expertise)?
    - Comments box (PMID)
- Databases and knowledgebases
    - Comments box (PMID)
- Reference materials, calibration, and normalization approaches
    - Comments box (PMID)
- Separation technologies (to be determined later)
    - Which EV separation technologies are mature enough to merit an ISEV guidelines or methods paper (list up to five with PMIDs to establish your expertise)?
    - Comments box (PMID)
- Therapeutics development and navigating regulatory affairs for therapeutics
    - Comments box (PMID)
